# Multiple Selected Changes May Modulate the Molecular Interaction between *Laverania* RH5 and Primate Basigin

**DOI:** 10.1128/mBio.00476-18

**Published:** 2018-05-22

**Authors:** Diego Forni, Chiara Pontremoli, Rachele Cagliani, Uberto Pozzoli, Mario Clerici, Manuela Sironi

**Affiliations:** a Scientific Institute IRCCS E.MEDEA, Bioinformatics, Bosisio Parini, Italy; b Department of Physiopathology and Transplantation, University of Milan, Milan, Italy; c Don C. Gnocchi Foundation ONLUS, IRCCS, Milan, Italy; Fred Hutchinson Cancer Research Center; Columbia University

**Keywords:** *Plasmodium falciparum*, RH5, adaptive evolution, basigin

## LETTER

Recently, Plenderleith et al. generated sequences of *Laverania* RH5 genes and analyzed their evolutionary history (1). Much of their efforts focus on criticism of a study in which we analyzed the evolution of RH5 (with much fewer sequences) and of primate basigin (BSG), the ligand for RH5 (2). Plenderleith et al. mistakenly quote sentences from our work and erroneously criticize our analyses (e.g., branch lengths were not “distorted,” they were simply not reported).

**FIG 1:**
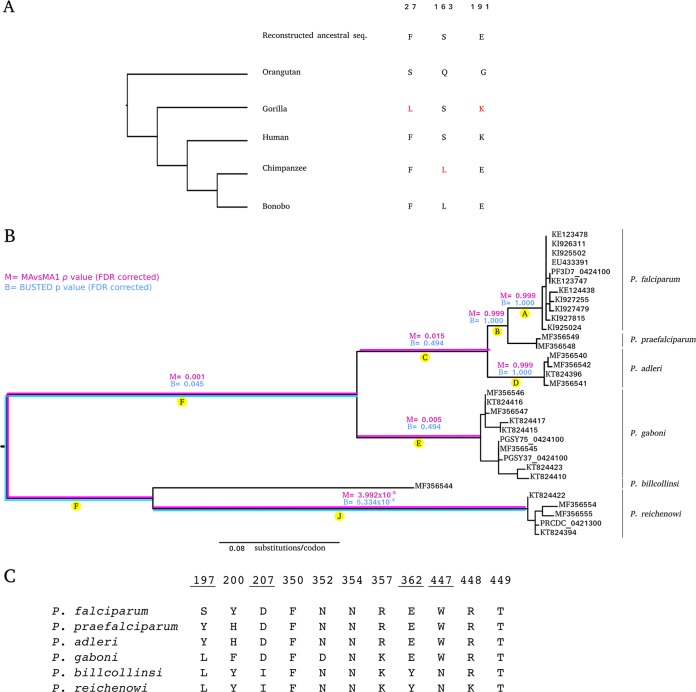
Positive selection at *RH5* and *BSG*. (A) BSG positively selected sites (shown in red) identified by Forni et al. (2) using a phylogenetics-population genetics method and located at the binding interface with PfRH5. The ancestral sequence was reconstructed using a maximum likelihood approach. (B) Phylogenetic tree of the *Laverania* species analyzed herein (sequence from Plenderleith et al. [1]). Letters denote branches tested as in reference 1. Branches showing statistical evidence of episodic positive selection with BUSTED (blue) and MA/MA1 (magenta) analyses are shown. False-discovery rate (FDR)-corrected *P* values are also reported. (C) Alignment of RH5 sites known to interact with BSG; the sites we found to be positively selected are underlined.

Notably, when Plenderleith et al. (1) mention our results on BSG, they fail to mention that, in addition to searching for evidence of selection in an extended primate phylogeny, we applied a phylogenetics-population genetics method (3) to search for sites that were positively selected in the human, chimpanzee, and gorilla lineages. Three of the sites we detected (Fig. 1) are located at the interaction surface with *Plasmodium falciparum* RH5 (PfRH5), and two of them strongly affect PfRH5 binding (4). When introduced into human BSG, the F27L change, which occurred in the gorilla lineage, causes an eightfold reduction in binding. As for residue 191, when the human amino acid (K) is introduced into the chimpanzee BSG protein (191E), the latter binds PfRH5 with much higher affinity (4). A conceivable interpretation of these findings is that chimpanzee and gorilla BSG evolved to avoid RH5 binding and, therefore, that positive selection at *BSG* contributed to determine species-specific interactions.

Concerning the analysis of RH5, we acknowledged in our work that we had little power to detect selection (see Discussion [2]), and we agree that we had no possibility to determine when selection acted. However, branch-site tests are robust to the inclusion of distantly related sequences and paralogs (5, 6). In fact, we identified two selected sites, one of which (position 447) was also detected by Plenderleith et al., who used BUSTED to search for selection across an RH5 *Laverania* phylogeny.

We have now applied two branch-site tests, the “MA/MA1” test and BUSTED to a phylogeny of almost complete RH5 sequences (Fig. 1). These methods allow the rigorous testing of *a priori*-specified branches for evidence of episodic positive selection. We used three methods to identify selected sites on branches showing evidence of selection (Fig. 1 and Table 1). Our results indicate the following. (i) The strongest selection occurred on the long branches that separate Plasmodium reichenowi/Plasmodium billcollinsi from the other *Laverania*. (ii) Selected sites include the two we had previously detected (190 and 447) and most of those described by Plenderleith et al. (1). (iii) No evidence of selection was detected on the P. falciparum branch. (iv) Selection at the 197 site, discussed by Plenderleith et al., most likely occurred in the ancestor of Plasmodium adleri and P. falciparum/Plasmodium praefalciparum. This clearly does not rule out the possibility that 197S in PfRH5 modulates binding.

**TABLE 1:** Positively selected sites detected in *Laverania* lineages

Branch^a^	Positively selected site(s) detected in *Laverania* lineage by^b^:		
	BEB	BUSTED	MEME
C			164, 174, 185, 197*, 346, 367, 380
E			122*, 228
F	447*	190, 207*, 361*, **362**, 447*	474
J	264, 323	263, 264, 271, 381*, 442*	263, 264, 271, 309, 381*, 442*

a Branches are named as by Plenderleith et al. (1).

b Sites under episodic positive selection found by Plenderleith et al. (1) are indicated with an asterisk. Sites identified by Forni et al. (2) are underlined. Sites involved in the interaction with BSG are shown in boldface type. The BEB posterior probability cutoff was 0.90, the BUSTED evidence ratio was >4, and the MEME *P* value cutoff was 0.1.

Analysis of *Laverania* RH5 amino acid residues at the BSG interaction surface indicates that, with the exclusion of position 197, no change occurred during P. falciparum speciation (Fig. 1). Thus, a quest for “the” PfRH5 variant responsible for the origin of P. falciparum as a human pathogen may prove unfruitful. However, we add that sites located distant from the interaction surfaces can affect binding properties (7) and that multiple changes often result in nonadditive effects of binding affinities (8).

Overall, we suggest that the RH5-BSG interaction should be viewed as a long-standing conflict in which multiple selected variants in both partners likely played a role.
